# Magnetosome organelles are organized through interactions between McaA and McaB that alter the dynamics of the bacterial actin-like protein MamK

**DOI:** 10.1128/mbio.00276-26

**Published:** 2026-04-03

**Authors:** Yein Ra, Yuanyuan Pan, Tansy Chen, Kayla Dixon, Azuma Taoka, Arash Komeili

**Affiliations:** 1 Department of Plant and Microbial Biology, University of California 118549, Berkeley, California, USA; 2 Institute of Science and Engineering, Kanazawa University 12858, Kanazawa, Ishikawa, Japan; Massachusetts Institute of Technology, Cambridge, Massachusetts, USA

**Keywords:** organelle assembly, biomineralization, magnetotactic bacteria, magnetosome, bacterial actin

## Abstract

Magnetotactic bacteria (MTB) are a group of gram-negative species that produce a lipid-bounded organelle, the magnetosome, in which a magnetic crystal is biomineralized. MTB use magnetosomes to align with the geomagnetic field for improved navigation of their environment. To optimize this alignment, these species linearly organize their magnetosomes using a handful of factors, including the bacterial actin-like protein MamK. Despite these shared features, there is a broad diversity of species-specific linear magnetosome chain arrangements within MTB, but the molecular mechanisms behind these phenotypic variations are unclear. Recently, genetic analyses showed that two proteins—McaA and McaB—interface with the chain organization machinery of *Magnetospirillum magneticum* AMB-1 to arrange magnetite crystals in a series of subchains rather than the single cohesive chains found in closely related MTB. Here, we use *in vivo* co-immunoprecipitation in AMB-1 to demonstrate protein-protein interactions between McaA, McaB, and MamK. Experiments with McaA truncation mutants and conditional control of McaB localization determined that McaA-McaB interactions are dependent on amino acids 530–665 of McaA and McaB localization to the magnetosome chain. We further show that disrupting the McaA-McaB interaction alters the spatial dynamics of MamK *in vivo*. We present a model in which protein-protein interactions between McaA, McaB, and MamK drive changes in MamK behavior to establish AMB-1’s magnetosome chain organization.

**IMPORTANCE:**

Magnetosomes serve as a model for understanding the cell biology of bacterial organelles and the mechanisms of bacterial cell organization. MamK, one of the best-studied bacterial actins, is a notable player in magnetosome chain assembly. This work explores how MamK dynamics are altered by two potential bacterial actin-binding proteins, McaA and McaB. This system illustrates how changes in bacterial cytoskeleton regulation result in different organization of subcellular compartments. The conclusions from this research also have implications for understanding the broader evolutionary strategies for the regulation of actin-like proteins and diversification of compartment organization in bacteria.

## INTRODUCTION

Similar to eukaryotes, many bacterial species produce one or more organelles that carry out a range of biochemical and behavioral activities (1–4). One of the best-studied bacterial organelles is the magnetosome (5). Produced by magnetotactic bacteria (MTB), these organelles start as a lipid-bounded compartment, wherein a magnetic crystal of magnetite (Fe_3_O_4_) or greigite (Fe_3_S_4_) is biomineralized (6, 7). Magnetosomes allow MTB to align with the Earth’s magnetic field, which is advantageous for navigating their aquatic environment with greater efficiency. Because MTB are typically motivated to find favorable oxygen environments, this behavior is termed magnetoaerotaxis (5). The ability to form magnetosomes makes MTB an attractive bacterial system to study biomineralization, organelle biogenesis, and compartment localization.

Magnetosomes exemplify how the function of an organelle can be closely tied to its subcellular localization. A prerequisite to magnetoaerotaxis is the organization of magnetosomes into a chain; various MTB mutants that produce magnetosomes but fail to organize them into a linear chain have dramatically lowered abilities to align with and navigate along a magnetic field (8–12). In the model MTB strains *Magnetospirillum magneticum* AMB-1 (hereafter AMB-1) and *Magnetospirillum gryphiswaldense* MSR-1 (hereafter MSR-1), the magnetosome chain has two important characteristics: it follows the positive curvature of the cell and is continuous, features that are controlled by MamY and MamK, respectively (13, 14). AMB-1 and MSR-1 maintain a linear magnetosome chain in a spiral-shaped body by positioning their chains along the positive cell curvature, thereby ensuring that the chain is parallel to the axis of cell motility. MamY assembles into a scaffold at the positive cell curvature onto which magnetosomes attach through a second protein, MamJ (14). The magnetosome chain is made continuous (i.e., there are no large gaps between magnetosomes) by MamK. MamK is a bacterial actin, and in its absence, the magnetosome chain is fragmented, not centered, and unevenly segregated during cell division (13, 15–19). Like eukaryotic actin, monomers of MamK polymerize into filaments when bound to ATP (20–22). Such MamK filaments are observed in AMB-1 and MSR-1 using cryo-electron tomography (9, 13). While within the filament, MamK monomers hydrolyze their ATP into ADP, favoring dissociation from the filament (20). The ATP-dependent polymerization and depolymerization of monomers are required for the dynamic quality and function of MamK. When MamK dynamics are perturbed by disrupting its ATPase activity, both chain assembly and magnetosome segregation are severely hindered (17). MamJ and its paralog LimJ connect magnetosomes to MamK filaments and are required for MamK dynamics in AMB-1 (9, 16, 23). MamJ is among the few known accessory factors of a bacterial actin, alongside ParR, AlfB, Alp7R, and others, which partner with distinct actin-like proteins to carry out diverse functions (24–26). Identifying additional proteins that regulate MamK can provide further insights into how bacterial cytoskeletons can be controlled.

**Fig 1:**
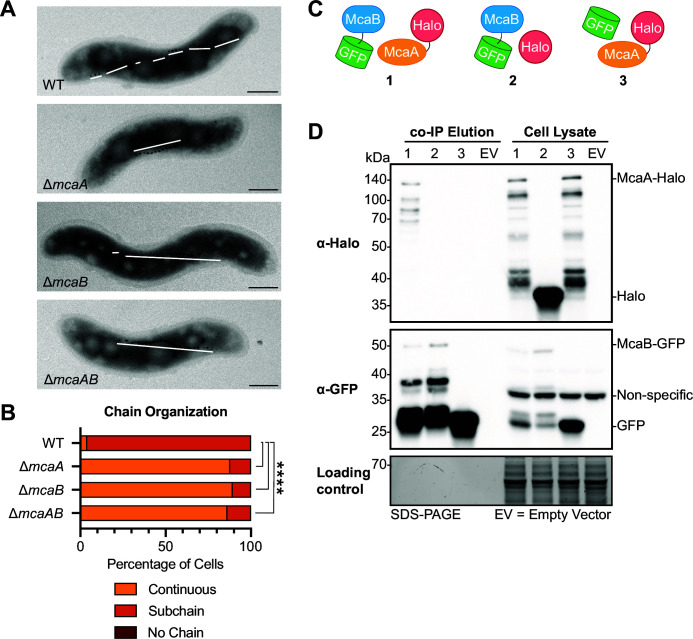
McaA and McaB contribute to magnetosome organization and interact *in vivo*. (**A**) Transmission electron microscopy images of a representative cell for AMB-1 WT, Δ*mcaA*, Δ*mcaB*, and Δ*mcaAB* showing the magnetosome chain. White lines are drawn to show the placement of crystal-containing magnetosomes. The WT magnetosome chain has many subchains of crystal-containing magnetosomes, while Δ*mcaA*, Δ*mcaB*, and Δ*mcaAB* do not. Scale bar = 0.5 µm. (**B**) Quantification of magnetosome chain organization using TEM images. Cells were categorized based on their magnetosome chain phenotype, and the *x*-axis represents the percentage of cells that displayed the indicated chain organization. WT *n* = 109, Δ*mcaA n* = 168, Δ*mcaB n* = 165, and Δ*mcaAB n* = 75. Fisher’s exact test was used to determine *P*-values (****P < 0.0001). (**C**) Schematics of the combination of proteins used for the *in vivo* co-IP in panel **D**. (**D**) Western blots of elutions from co-IP with GFP-Trap agarose beads and cell lysates illustrating that McaA-Halo and McaB-GFP interact, and this interaction is not driven by their tags. The co-IP was done using the Δ*mcaAB* strain. The loading control is a stain-free SDS-PAGE gel of the same samples. This result is a representative of three biological replicates (see Fig. S3).

Recently, two additional proteins that alter MamK behavior have been identified. In AMB-1 strains missing *mcaA* and *mcaB*, MamK dynamics are changed when observed by fluorescence recovery after photobleaching (FRAP). Additionally, McaA and McaB are required for AMB-1’s magnetosome chain organization. At first glance, with transmission electron microscopy (TEM), the WT AMB-1 magnetosome chain appears fragmented (Fig. 1A). Imaging with cryo-electron tomography, however, reveals that the gaps between crystal subchains are populated with magnetosome membranes that have not yet initiated crystal biomineralization (10). Thus, AMB-1 magnetosomes are organized in subgroups of crystal-containing magnetosomes or empty magnetosomes that together form a continuous chain. In the absence of *mcaA*, *mcaB*, or both, the mutant chain resembles that of WT MSR-1, where magnetite crystals are continuous and at midcell while empty magnetosomes are at the chain periphery (Fig. 1A and B) (9, 10, 14). This has collectively led to the hypothesis that McaA and McaB create a magnetosome chain with crystal subchains by influencing MamK behavior (10). Investigating how the Mca proteins contribute to magnetosome chain organization will resolve the mechanism behind how AMB-1 and MSR-1, two organisms that share ~96% identity in their 16S rRNA genes (27), pattern their magnetosomes in a strikingly different manner. Currently, how McaA and McaB work together at the protein level, and whether they directly affect MamK, is unknown.

Here, we discovered that McaA, McaB, and MamK form protein-protein interactions with each other. The McaA-McaB interaction is mediated through a conserved region on McaA and primarily occurs when McaB is localized to the magnetosome chain. Disrupting the McaA-McaB interactions by truncating McaA or altering McaB localization is accompanied by a shift in MamK dynamics. Altogether, the protein-protein interactions of McaA, McaB, and MamK affect MamK dynamics and ultimately organize magnetic crystals into subchains in AMB-1.

## RESULTS

### McaA and McaB interact with each other

The genetic studies of *mcaA* and *mcaB* suggest that there is a machinery dedicated to producing the crystal subchain magnetosome organization (10). To gain insight into the mechanism behind this magnetosome positioning system, we set out to determine which proteins interact with McaA and McaB *in vivo* in AMB-1. We separately expressed GFP-tagged McaA and McaB on replicative plasmids in AMB-1, which have previously been shown to complement their respective gene deletions (10), and used GFP-trap agarose beads for co-immunoprecipitation paired with mass spectrometry (IP-MS). The experiment was also performed with GFP alone to determine which interactions were due to the tag or non-specific binding to the agarose beads (Data S2).

We first attempted to identify other magnetosome proteins involved in magnetosome chain organization by looking for proteins that co-eluted with both McaA-GFP and McaB-GFP. The McaB-GFP IP-MS results were poor due to low protein abundance, but we still identified peptides from four magnetosome proteins: MamA, MamQ, MamJ, and Mms6 (Data S2). MamA and MamQ were also identified in the IP-MS with McaA-GFP. To assess whether MamA has a role in magnetosome chain organization, the *mamA* deletion strain was imaged with TEM. No deviation in magnetosome chain organization was observed (Fig. S1A and B), suggesting that MamA is not necessary for the crystal subchain phenotype. Because AMB-1 Δ*mamQ* does not produce magnetosomes, magnetosome arrangement could not be assessed in this strain.

The IP-MS results from McaA-GFP also revealed that McaA and McaB may interact in AMB-1 (Data S2). To verify this result, the co-immunoprecipitation (co-IP) using GFP-trap beads was repeated in triplicate with a Δ*mcaAB* strain expressing McaB-GFP and McaA-Halo. These tagged proteins can complement their respective deletion when assessed by TEM and coefficient of magnetism (*C*_mag_) measurements (Fig. S2), which measures the degree of magnetic field alignment by MTB cells in a liquid culture (7). The elution was probed for the presence of both proteins using Western blotting. Both proteins co-eluted (Fig. 1C and D; Fig. S3A), indicating that indeed, McaA and McaB interactions occur in AMB-1. Co-IPs with McaB-GFP/Halo and GFP/McaA-Halo pairs expressed in Δ*mcaAB* confirmed that the GFP and Halo tags, or nonspecific interactions with beads, were not responsible for the interaction (Fig. 1D; Fig. S3B). Samples containing McaA-Halo almost always had a series of smaller bands in the anti-Halo Western blot (Fig. 1D). These appear to be specific to McaA-Halo, since they are not present in samples expressing other Halo-tagged proteins (Fig. 1D; Fig. S3B, S8D, and S13C). These bands could represent McaA-Halo degradation products or be a result of post-translational processing of McaA-Halo. In the anti-GFP blots of McaB-GFP samples, there is a strong signal from a protein that is around the size of GFP (26.8 kDa). In the *mcaB-gfp* fusion, the start codon of GFP was not removed. Thus, there might be an internal ribosome-binding site that drives the translation of free GFP. Given that our negative control co-IP with free GFP and McaA-Halo does not detect an interaction (Fig. 1D; Fig. S3B), it is unlikely that this free GFP is driving the interaction in the McaA-Halo/McaB-GFP co-IP.

Having observed McaA-McaB interactions in AMB-1, we investigated whether the interaction between them was direct using purified proteins. Because McaA and McaB are both predicted to contain transmembrane domains (10), the domain architectures of both proteins were closely studied to identify a soluble, cytoplasmic portion that can be purified with minimal impact on potentially important domains (see Fig. 3A and B). Analysis with DeepCoil2 revealed a high probability that McaB amino acids 81–112 form a coiled-coil domain (Fig. S4A) (28). Coiled-coil domains are alpha-helices that oligomerize by burying their hydrophobic residues, commonly referred to as “a” and “d” position amino acids, against each other. We mapped the “a” and “d” position amino acids predicted from DeepCoil2 onto the AlphaFold3-predicted McaB monomer, dimer, and trimer (Fig. S4B through D) (29). We found that in a trimer, the “a” and “d” position residues are indeed predicted to form a hydrophobic core (Fig. S4D). Truncating the N-terminal end up to the transmembrane domain of McaB (amino acids 1–27) was not predicted by Alphafold to disrupt this coiled-coil structure (Fig. S4E).

McaA is also predicted to contain a transmembrane domain (10). It has a predicted periplasmic signal peptide and von Willebrand factor type A (VWA) domain at its N-terminus (see Fig. 3A) that has been previously demonstrated to be necessary for its localization to the positive inner curvature of the cell (10). Within the C-terminal cytoplasmic side of the transmembrane domain (aa392 and onward), McaA has a stretch of amino acids from 530 to 665 (named here the “530 region”) that is required for its function in magnetosome organization (10). Given that McaB has only four amino acids predicted to be on the N-terminal side of its transmembrane domain (see Fig. 3B), it seemed unlikely that McaA and McaB interact at the periplasm or magnetosome lumen. We thus reasoned that domains necessary for interaction with McaB and McaA function are on the cytoplasmic side of McaA. Therefore, McaA aa400–776 (McaA_cyto_) and McaB aa28–219 (McaB_cyto_) were chosen to be purified.

**Fig 2:**
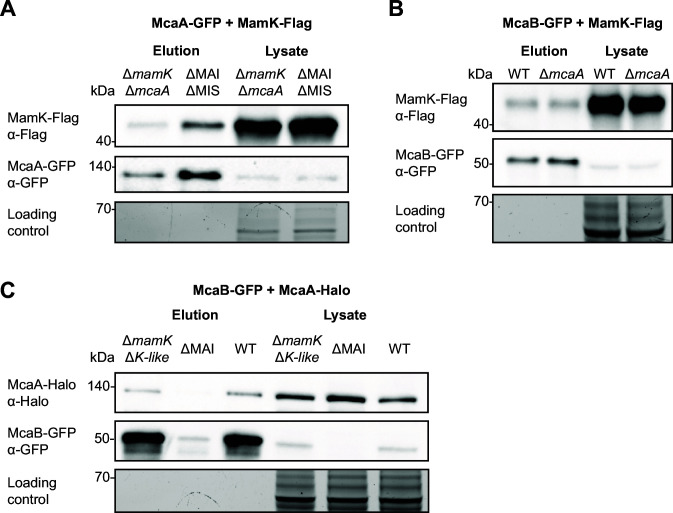
MamK interacts with both McaA and McaB *in vivo*. (**A**) Western blots of a co-IP with McaA-GFP and MamK-Flag were done in both Δ*mamK*Δ*mcaA* and ΔMAIΔMIS. (**B**) Western blots of a co-IP with McaB-GFP and MamK-Flag in WT and Δ*mcaA*. (**C**) Western blots of a co-IP with McaB-GFP and McaA-Halo were done in Δ*mamK*Δ*mamK-like*, ΔMAI, and WT. In all cases, GFP-Trap agarose beads were used for co-IPs, and a stain-free SDS-PAGE serves as the loading control.

To that end, we constructed McaA_cyto_ with an N-terminal Strep-tag and McaB_cyto_ with an N-terminal maltose-binding protein (MBP) tag and purified them using affinity chromatography and size exclusion chromatography (Fig. S5A). We performed *in vitro* pull-downs with Strep-McaA_cyto_ and MBP-McaB_cyto_ using streptavidin beads. If there is an interaction between them, then both proteins should be found in the elution fraction. However, the elution only contained Strep-McaA_cyto_ (Fig. S5B). Similarly, bacterial two-hybrid assays did not detect an interaction between full-length McaB and full-length McaA or McaA_cyto_ (10) (Fig. S6B). It is possible that the interaction between the two proteins requires proper localization to the membrane. Alternatively, the McaA-McaB interaction observed *in vivo* might be bridged by one or more proteins. To test whether any magnetosome proteins fulfill this role, we attempted to repeat the McaB-GFP/McaA-Halo co-IP in AMB-1 ΔMAI, a strain missing the magnetosome island (MAI) that encodes the majority of AMB-1 magnetosome genes (30). However, McaB-GFP is expressed poorly or unstable in this strain, as indicated by a weak signal in the lysate anti-GFP Western blot (Fig. 2C; Fig. S8A), perhaps because an MAI-encoded protein is required for McaB expression or stability. Thus, we were unable to assess the interaction in this background.

### MamK interacts with McaA and McaB *in vivo*

McaA-GFP IP-MS data showed that MamK may also interact with McaA. As discussed above, MamK is a bacterial actin that is responsible for assembling magnetosomes into a linear chain (13, 17). Furthermore, MamK dynamics are altered in Δ*mcaA* and Δ*mcaB* strains (10). A co-IP with McaA-GFP and MamK-Flag confirmed the interaction between these two proteins (Fig. 2A; Fig. S7A). Co-IPs with GFP/MamK-Flag and McaA-GFP/Halo-Flag combinations ensured that the observed interactions were not due to the GFP or Flag tags (Fig. S7A). To address whether this interaction is bridged by any other magnetosome protein, including McaB, we assessed whether McaA-GFP and MamK-Flag would interact in ΔMAIΔMIS, a strain missing the magnetotactic islet (MIS) in addition to the MAI (10, 31). We found that MamK-Flag still elutes with McaA-GFP, signifying that the McaA-MamK interaction does not require other magnetosome proteins (Fig. 2A; Fig. S8B).

Since the signal from the McaB-GFP IP-MS experiment was poor, we also considered the possibility that an McaB interaction with MamK might have been missed. We therefore checked for McaB-MamK interactions using co-IP with McaB-GFP and MamK-Flag. Indeed, the two proteins co-eluted (Fig. 2B; Fig. S7B). This interaction is also independent of McaA, as the two proteins still interacted in an *mcaA* deletion background (Fig. 2B; Fig. S8C). The interaction is also not driven by the GFP and Flag tags, since co-IP with GFP/MamK-Flag and McaB-GFP/Halo-Flag in a Δ*mcaB*Δ*mamK* background did not result in co-elution of the protein pairs (Fig. S7B). The McaB-MamK interaction was not tested in ΔMAIΔMIS since we previously found McaB expression to be unstable in strains missing the MAI (Fig. 2C; Fig. S8A). Finally, we asked whether MamK is necessary for the McaA-McaB interaction. We repeated the McaB-GFP and McaA-Halo co-IP in AMB-1 Δ*mamK*Δ*mamK-like*, a double mutant missing both *mamK* and its homolog *mamK-like* that is located within the MIS (10, 31). McaB-GFP was stable in this background and interacted with McaA-Halo (Fig. 2C; Fig. S8A). In summary, we used a series of co-IP experiments to show that McaA, McaB, and MamK all have independent pairwise interactions with one another in AMB-1.

### MamK directly interacts with McaA and McaB

**Fig 3:**
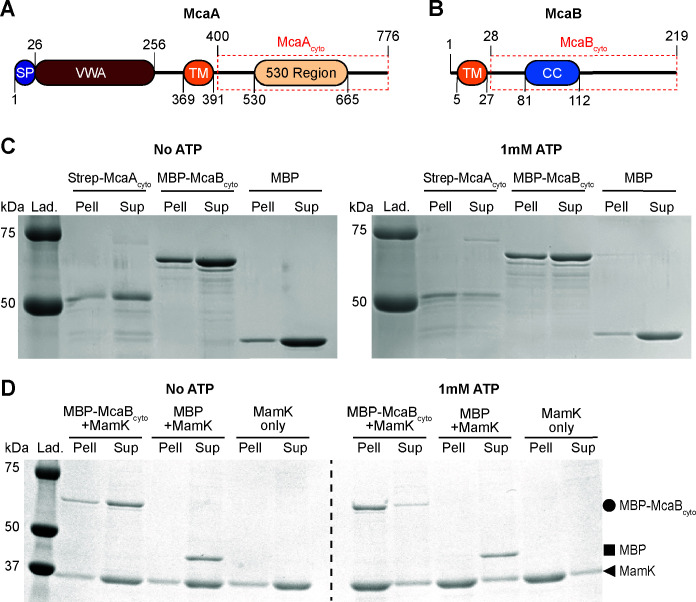
Purified McaB_cyto_ interacts with MamK filaments. (**A**) Schematic of McaA showing the predicted signal peptide (SP), von Willebrand factor type A (VWA) domain, transmembrane domain (TM), and the conserved 530 region. The amino acid positions are indicated, and the red dashed box represents the segment purified as McaA_cyto_. (**B**) Schematic of McaB showing the predicted transmembrane domain (TM) and coiled-coil domain (CC). The amino acid positions are indicated, and the red dashed box represents the segment purified as McaB_cyto_. (**C**) SDS-PAGE of pelleting assay samples. Proteins were incubated with or without 1 mM ATP and then separated into the pellet (Pell) and supernatant (Sup) fractions. (**D**) SDS-PAGE of co-pelleting assay samples. MamK was mixed with either MBP-McaB_cyto_ or MBP and incubated with or without 1 mM ATP. The sample was separated into the pellet (Pell) and supernatant (Sup) fractions. MamK polymerizes into filaments in the presence of ATP, demonstrated by the shift into the pellet fraction when incubated with ATP. MBP-McaB_cyto_ shifts into the pellet fraction in the presence of MamK filaments, indicating an interaction between MBP-McaB_cyto_ and MamK. MBP alone remains predominantly in the supernatant in the parallel experiment. MBP-McaB_cyto_ (65.0 kDa), MBP (43.2 kDa), and MamK (37.6 kDa) bands are indicated with a circle, square, and triangle, respectively. Lanes with protein ladders (Lad.) are also included.

The protein-protein interactions between McaA, McaB, and MamK, paired with the previously observed effect of McaA and McaB on MamK dynamics, raised the possibility that the Mca proteins are direct regulators of MamK behavior. To investigate direct interactions between these proteins, untagged recombinant MamK was expressed in *Escherichia coli* BL21 and purified using ammonium sulfate precipitation and size exclusion chromatography (Fig. S5A). Purified MamK was tested for interactions against recombinant McaA_cyto_ and McaB_cyto_ *in vitro*. MamK forms filaments when bound to ATP, which can be separated from MamK monomers using a pelleting assay (Fig. 3D). We used this assay to assess whether McaA or McaB co-pellets with MamK filaments. MBP-McaB_cyto_ co-pelleted with MamK in an ATP-dependent manner while the MBP tag alone predominantly remained in the supernatant (Fig. 3D), signifying that McaB_cyto_ directly binds MamK filaments. Strep-McaA_cyto_ pellets on their own with ATP (Fig. 3C). Therefore, we could not use this assay to determine its direct interaction with MamK. We instead turned to bacterial two-hybrid assays, which detected an interaction between McaA_cyto_ and MamK (Fig. S6A). This interaction was missed in a previous bacterial two-hybrid assay where LB medium was used instead of M63 (10). Thus, MamK interacts with both McaA and McaB directly as observed by pelleting and bacterial two-hybrid assays.

### Interaction between McaA and McaB is mediated through a conserved domain of McaA

**Fig 4:**
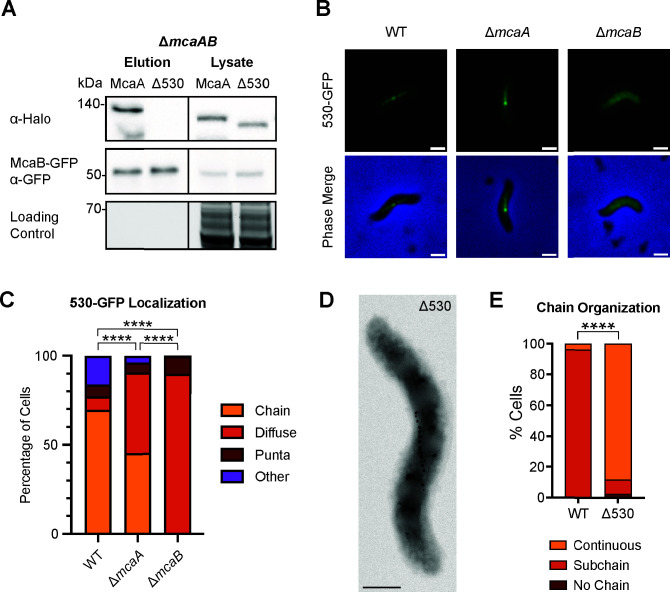
The 530 region of McaA is necessary for interaction with McaB and the crystal subchain phenotype. (**A**) Western blots of co-IPs with McaB-GFP and either full-length McaA-Halo or McaA-Halo missing the 530 region (Δ530) in a Δ*mcaAB* strain using GFP-Trap agarose beads. The loading control is a stain-free SDS-PAGE gel. (**B**) The 530 region fragment was tagged with GFP and expressed in WT, Δ*mcaA*, and Δ*mcaB* and then imaged with epifluorescence microscopy. Representative cells for each expression strain are shown. Scale bar = 1 µm. (**C**) Quantification of 530-GFP localization from fluorescent microscopy images. Cells were categorized based on their localization pattern, and the *y*-axis represents the percentage of cells that displayed the indicated localization. WT *n* = 390, Δ*mcaA n* = 390, and Δ*mcaB n* = 332. Fisher’s exact test was used to determine *P*-values (****P < 0.0001). (**D**) Representative TEM image of AMB-1 with the 530 region deleted at the native locus. Scale bar = 0.5 µm. (**E**) Quantification of magnetosome chain organization using TEM images. Cells were categorized based on their magnetosome chain phenotype, and the *y*-axis represents the percentage of cells that displayed the indicated chain organization. WT *n* = 75, Δ530 *n* = 77. Fisher’s exact test was used to determine *P*-values (****P < 0.0001).

Next, we asked whether disrupting the McaA-McaB interaction would have an impact on their function *in vivo*. To that end, we searched for possible domains necessary for the McaA-McaB interaction. The 530 region (aa530–665) of McaA is more conserved compared to the rest of the C-terminal side (Fig. S9B) and required for WT magnetosome chain organization. It has been shown that McaA without the 530 region (McaAΔ530) is unable to complement the *mcaA* deletion; Δ*mcaA* strain expressing McaAΔ530-GFP on a plasmid still produces a continuous chain of magnetite crystals despite having the same localization as full-length McaA-GFP (10). McaAΔ530-GFP is also the only truncation made on the cytoplasmic side of McaA that cannot complement the Δ*mcaA* deletion (10). Guided by Alphafold3 structure predictions (29), the 530 region was further divided into three smaller segments, and each was removed from McaA-GFP (Fig. S10A). Each GFP-tagged truncation had the same localization pattern as full-length McaA-GFP (Fig. S10F). However, none of the three truncated McaA-GFP restored the crystal subchain magnetosome organization when expressed in the *mcaA* deletion, and the *C*_mag_ of these strains was similar to that of Δ*mcaA* with an empty vector (Fig. S10C through E). Thus, the entire 530 region is necessary for McaA’s function. We hypothesized that the loss of function of McaAΔ530 could be due to loss of interaction with McaB or MamK. To test this, we expressed McaB-GFP and McaAΔ530-Halo in Δ*mcaAB* and tested for interaction between the proteins using a co-IP assay. Indeed, McaAΔ530-Halo did not elute with McaB-GFP (Fig. 4A; Fig. S3A). On the other hand, McaAΔ530-GFP retained its interaction with MamK-Flag in Δ*mcaA*Δ*mamK* and ΔMAIΔMIS strains (Fig. S7A and S8B). Thus, the 530 region is necessary for McaA function, and its interaction with McaB, and the loss-of-function of McaAΔ530 is not due to a lack of interaction with MamK.

To determine whether the 530 region is sufficient for interaction with McaB, we cloned a fragment of McaA spanning this region and tagged it with GFP (530-GFP). We then expressed this construct in WT, Δ*mcaA*, and Δ*mcaB*. In WT, the majority of cells displayed 530-GFP with a magnetosome chain localization (Fig. 4B and C). This localization is not dependent on full-length McaA; though there was an increase in cells with a diffuse 530-GFP signal, a large proportion of the population still had 530-GFP localized to the magnetosome chain (Fig. 4B and C). This magnetosome chain localization is almost completely lost in the absence of *mcaB*; the vast majority of Δ*mcaB* cells have a diffuse 530-GFP signal (Fig. 4B and C). One explanation for this is that the 530 region is sufficient to interact with McaB, and this interaction draws 530-GFP to the magnetosome chain. To confirm this claim, we conducted a co-IP using the Δ*mcaAB* strain expressing McaB-GFP and a Halo-tagged 530 region (530-Halo) (Fig. S8D). Indeed, the 530-Halo co-eluted with McaB-GFP, signifying that the 530 region is sufficient for interaction with McaB.

### McaA 530 region is necessary for WT MamK dynamics

It has been previously shown that MamK dynamics are impacted by McaA and McaB. In prior FRAP experiments with WT AMB-1 expressing MamK-GFP, the photobleached region was always static and recovered in place (10). In Δ*mcaA* and Δ*mcaB*, on the other hand, the photobleached region was motile in a portion of the cells, indicating that MamK dynamics is influenced by the Mca proteins (10). We hypothesized that the interaction between McaA and McaB, which appears critical for magnetosome chain patterning, also contributes to the dynamics of MamK recovery in FRAP.

**Fig 5:**
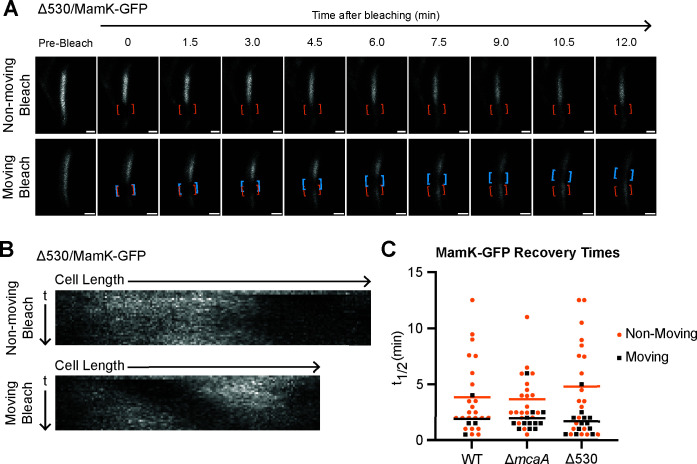
The 530 region is required for WT MamK dynamics. (**A**) Two representative cells from FRAP of MamK-GFP in Δ530 strain that were imaged using confocal microscopy. Orange brackets indicate the original bleaching location. Blue brackets track bleach spot movements. The top cell has a bleach spot that does not move, while the bottom cell has a moving bleach spot. Scale bar = 0.5 µm. (**B**) Kymographs of the cells in panel **A**. (**C**) MamK-GFP half-time of fluorescence recovery (t_1/2_) across different AMB-1 strains. Each point represents one cell. Yellow circles indicate cells with non-moving bleach spots, while black triangles represent those with a moving bleach spot. Cells that did not recover were not included. Bars represent the average recovery half-time of each population.

To test this, we first made an in-frame deletion of the 530 region at the native *mcaA* locus. Like Δ*mcaA*, this strain has a magnetosome chain with crystal-containing magnetosomes at the chain middle (Fig. 4D and E), and its *C*_mag_ is higher than that of WT (Fig. S10B). We then expressed MamK-GFP in WT, Δ*mcaA*, and Δ530 and observed MamK dynamics using FRAP. We found MamK-GFP to be dynamic in all strain backgrounds (Fig. 5A through C; Fig. S11). We first compared the half-time of fluorescence recovery (*t*_1/2_), the time it takes for the bleached region to regain 50% fluorescence intensity of the whole filament intensity (Table 1). When examining the entire population of recovered cells across WT, Δ*mcaA*, and Δ530, the *t*_1/2_ did not substantially vary (3.53 ± 3.15 min, 3.02 ± 2.22 min, 3.65 ± 3.67 min, respectively). In most WT cells that recovered, the bleach spot was stationary and recovered in place. However, the bleach spot moved during recovery in about 15% of WT cells observed. In a Δ*mcaA* strain, the number of cells with a MamK-GFP moving bleach spot more than doubled (38% of recovered cells observed), consistent with prior observations (10). Similarly, 38% of recovered Δ530 cells exhibited the moving bleach spot pattern (Fig. 5A and B). MamK-GFP behavior thus shifted more toward moving-bleach-spot dynamics in Δ530, as in Δ*mcaA* (Fig. 5C). When we separated the recovered cell population based on MamK recovery behavior (non-moving vs. moving bleach spot), we can see that, in general, cells with a moving bleach spot recover faster than those with non-moving bleach spots (Table 1). Recovery times were independent of cell length, with the exception of Δ530 cells, in which those with non-moving bleach spots showed a slight positive correlation (Fig. S12A). The directions of the moving bleach spots were not consistent from cell to cell; the moving bleach spot moved toward the cell pole in some cells and towards midcell in others (Fig. S12B). Altogether, the change in magnetosome organization and altered MamK dynamics correlate with the loss of the McaA-McaB interaction.

**TABLE 1:** MamK-GFP dynamics observed by FRAP

Strain	*n*	% recovered	% moving bleach of recovered	*t*_1/2_ (average ± SD [min])		
				All cells	Non-moving bleach cells	Moving bleach cells
WT/MamK-GFP	31	87 (*n* = 27)	15 (*n* = 4)	3.53 ± 3.15	3.82 ± 3.29	1.87 ± 1.50
ΔmcaA/MamK-GFP	32	100 (*n* = 32)	38 (*n* = 12)	3.02 ± 2.22	3.65 ± 2.41	1.95 ± 1.37
Δ530/MamK-GFP	35	91 (*n* = 32)	38 (*n* = 12)	3.65 ± 3.67	4.79 ± 4.14	1.66 ± 1.24
WT/MamK-GFP, nonbiomin	32	78 (*n* = 25)	36 (*n* = 9)	5.11 ± 3.56	5.06 ± 3.64	5.21 ± 3.63

### Non-biomineralization conditions affect McaB-GFP localization, McaA-McaB interaction, and MamK dynamics

McaAB-mediated change of MamK dynamics appears to be dependent on McaA-McaB interactions. This led us to consider that MamK behavior might be locally controlled based on where McaA-McaB interactions occur along the magnetosome chain. We further speculated that McaA-McaB interactions are limited to where the two proteins co-localize. To assess the role of localization on McaA-McaB interactions, we perturbed McaB localization to crystal-containing magnetosomes.

McaB’s localization pattern resembles that of another magnetosome protein, Mms6. Mms6 is involved in the biomineralization of the magnetite crystal, and like McaB, is present on magnetosomes that contain magnetite (32, 33). Furthermore, Mms6 was identified in the IP-MS with McaB-GFP. Therefore, we hypothesized that McaB is recruited to crystal-containing magnetosomes through an interaction with Mms6. We tested this by comparing McaB-GFP localization in WT and Δ*mms6*. At first glance, the McaB-GFP signal seems diffuse in Δ*mms6* compared to WT. However, upon closer inspection, we noted that a subpopulation of McaB-GFP is still found at the magnetosome chain in most cells (Fig. S13A and B). Additionally, we could not confirm the Mms6-McaB interaction through co-IPs with Mms6-Halo and McaB-GFP in WT AMB-1 (Fig. S13C). A co-IP of McaB-GFP and McaA-Halo determined that the two proteins still interact in Δ*mms6* (Fig. S13D), and TEM images of AMB-1 Δ*mms6* revealed only a minimal change in magnetosome arrangement in this strain (Fig. S13E and F). Therefore, Mms6 is not necessary for recruitment of McaB to the magnetosome chain, the McaA-McaB interaction, and proper chain formation.

**Fig 6:**
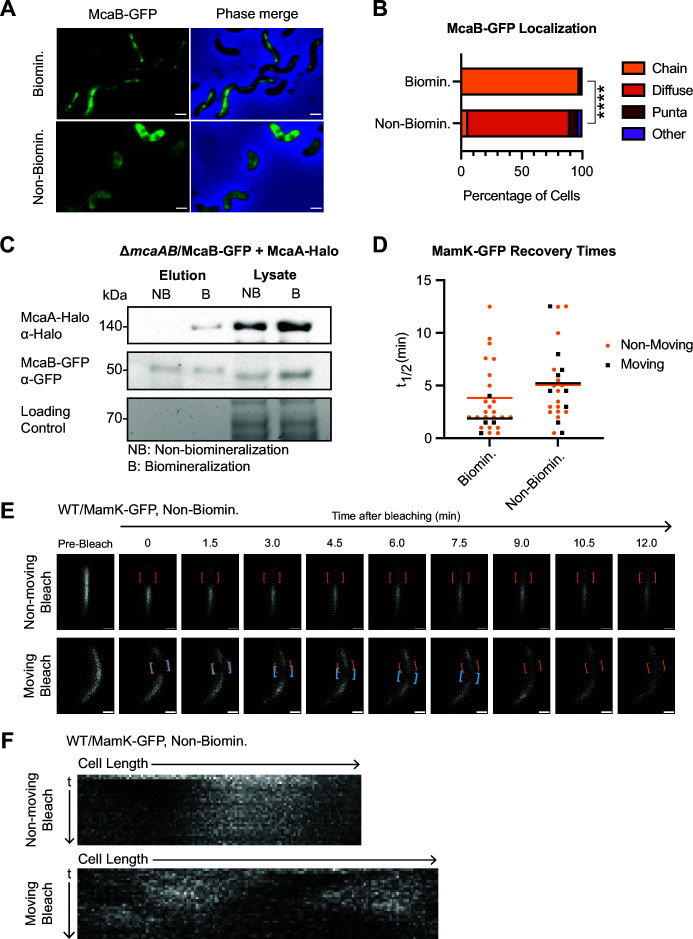
Changes to McaB localization correlate with its ability to interact with McaA and shift in MamK dynamics. (**A**) Representative cells showing McaB-GFP localization in biomineralization and non-biomineralization conditions in the same strain used for co-IP in panel **C**. Images were taken with epifluorescence microscopy. Scale bar = 1 µm. (**B**) Quantification of McaB-GFP localization using fluorescent microscopy images. Cells were categorized based on their localization pattern, and the *y*-axis represents the percentage of cells that displayed the indicated chain organization. Biomineralization *n* = 845, non-biomineralization *n* = 4,243. Chi-squared test of independence was used to determine *P*-values (****P < 0.0001). (**C**) Western blots of co-IP with McaB-GFP and McaA-Halo in non-biomineralization (NB) and biomineralization (B) conditions in a Δ*mcaAB* background using GFP-Trap agarose beads. The loading control is a stain-free SDS-PAGE gel. (**D**) Recovery half-times (*t*_1/2_) of MamK-GFP in WT AMB-1 under biomineralization (same as WT from Fig. 5C) or non-biomineralization conditions. Each point represents one cell. Yellow dots indicate cells with a non-moving bleach spot, while black squares represent those with a moving bleach spot. Cells that did not recover were not included. Bars represent the average recovery half-time of each population. (**E**) Two representative cells from FRAP of MamK-GFP in WT grown under non-biomineralizing conditions imaged using confocal microscopy. Orange brackets indicate the original bleaching location. Blue brackets track bleach spot movements. The top cell has a bleach spot that does not move, while the bottom cell has a moving bleach spot. Scale bar = 0.5 µm. (**F**) Kymographs of the same cells depicted in panel **E**.

We next turned to previous studies that showed a conditional change in McaB-GFP localization. When AMB-1 cells are shifted to an iron-limited growth medium, they produce a chain of magnetosome membranes that are devoid of magnetite crystals (7, 30). In previous work, we found that McaB-GFP localization appears diffuse under these non-biomineralizing conditions (10). We first quantified the difference in McaB-GFP localization in both biomineralization and non-biomineralization conditions in WT AMB-1. Indeed, in non-biomineralization growth, the majority of AMB-1 cells show diffuse McaB-GFP signal (Fig. 6A and B). We then tested whether McaB still interacts with McaA when it is no longer localized to the magnetosome chain. Compared to biomineralizing conditions, McaB interactions with full-size McaA are notably reduced in non-biomineralizing conditions (Fig. 6C). Interestingly, shorter McaA-Halo bands still co-eluted with McaB-GFP (Fig. S14A, Source Data). We speculate that some degradation products of McaA-Halo have lost their transmembrane domain. This soluble McaA-Halo truncation can then interact with the diffuse McaB-GFP, which further implies that protein localization, not growth condition, is the key contributor to the McaA-McaB interaction. Altogether, McaB localization to the magnetosome chain is a prerequisite for its interaction with McaA.

Since McaA-McaB interactions are missing in non-biomineralizing conditions, we predicted that MamK dynamics would also be impacted. We subjected WT AMB-1-expressing MamK-GFP to non-biomineralization conditions and then conducted FRAP. We found that in general, MamK-GFP recovery was much slower when cells were grown in non-biomineralization conditions with an average *t*_1/2_ of 5.11 ± 3.56 min, and 22% of cells did not recover in the observed timeframe (Table 1). The longer recovery times may reflect a broader metabolic constraint from growing in iron-limited medium, demonstrated by the decreased growth observed under non-biomineralization conditions (Fig. S14B). Despite the overall increase in *t*_1/2_, we observed that 36% of recovering cells displayed the moving-bleach-spot dynamics, similar to Δ*mcaA* and Δ530 (Fig. 6D through F; Table 1). This is consistent with our hypothesis that the effect of McaA and McaB on MamK dynamics is dependent on their ability to interact with each other, which is in part driven by McaB’s localization to the magnetosome chain.

## DISCUSSION

### McaA-McaB interactions drive magnetosome organization through MamK dynamics

In this study, we set out to determine how McaA and McaB produce the subchain organization of magnetosomes in AMB-1. Through both *in vivo* and *in vitro* work, we discovered that McaA and McaB have protein-protein interactions with each other and the bacterial actin MamK. We further demonstrated that each pairwise interaction occurs independently of the third protein. Disrupting one of these interactions (McaA-McaB) leads to a change in magnetosome organization from subchains of magnetite crystals to a singular crystal chain. This points to a model where a network of McaA, McaB, and MamK interactions is required for magnetosome chain organization.

**Fig 7:**
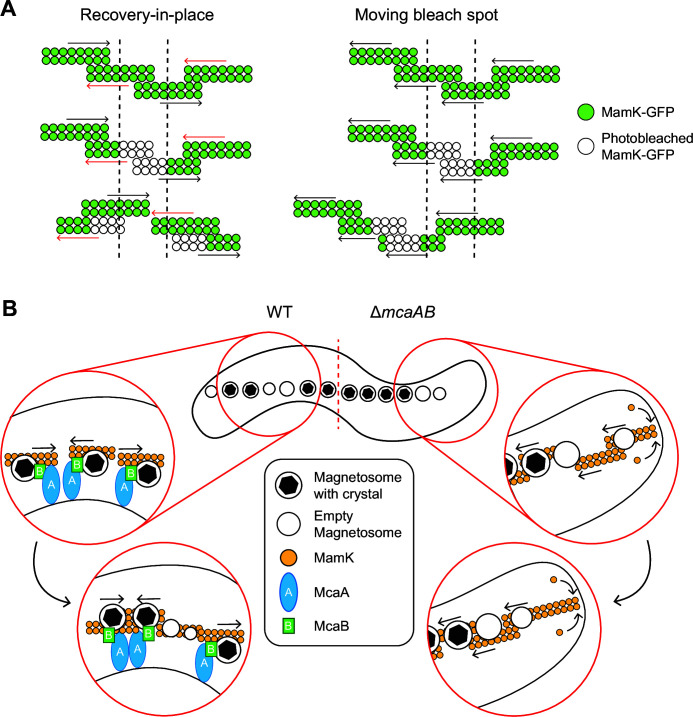
Model for how the magnetosome chain organization is established in WT AMB-1. (**A**) Schematic displaying how the dynamics of MamK filaments produced the two observed FRAP behaviors. Green circles depict fluorescent MamK-GFP, while white circles represent photobleached MamK-GFP during the FRAP experiments. The dashed lines indicate the location of photobleaching. In situations where the MamK-GFP bleach spot does not move and is recovering in place (left), nearby MamK filaments are moving or polymerizing in either direction, represented by the arrows. The bleached filaments may move away while unbleached filaments move into the region, causing the gradual recovery of the bleached location in place. In cells where the bleach spot is moving (right), nearby MamK filaments have coordinated movement in a single direction. The bleach spot also recovers during its movement, likely because the individual filaments undergo polymerization while moving. (**B**) Model for how McaA and McaB produce crystal subchains by altering MamK dynamics in AMB-1. In WT (left), McaA-McaB interactions occur along the magnetosome chain where crystal-containing magnetosomes are present. This allows the McaAB complex to interact with MamK filaments to alter their directionality, resulting in subgroups of crystal-containing magnetosomes. Concentrating crystal-containing magnetosomes into subchains creates space throughout the cell for new magnetosomes to form. In the absence of *mcaAB* (right), MamK filaments move in a single direction, pushing magnetosomes to the midcell. The oldest magnetosomes—most of which have crystals—are at the middle of the chain and the newest are at the chain periphery.

The effect of McaAB on magnetosome organization is closely tied to MamK dynamics. Two types of MamK dynamics were observed through our FRAP imaging: moving-bleach-spot dynamics, where the bleach spot migrated from its original position while recovering, and recovery-in-place dynamics, where the bleach spot recovered without moving. It is challenging to extract the behavior of individual MamK filaments from this data because, during FRAP, many overlapping 200 nm sized MamK filaments are simultaneously photobleached (13). Consequently, our interpretation of the FRAP data is focused on bulk MamK filament behavior. We predict that the moving-bleach-spot dynamic is due to a global organization of individual MamK filaments in which the majority of MamK filaments move or polymerize in the same direction (Fig. 7A). We currently do not know how the movement is coordinated or how the movement direction is determined. In contrast, the recovery-in-place dynamics is perhaps a result of individual MamK filaments moving or polymerizing in either direction along the cell length (Fig. 7A). Though the directionality of individual filaments might be regulated locally, the movement direction is not uniform throughout the entire population of MamK filaments. Interestingly, MamK dynamics are not identical within an entire population. Even in populations that had more cells with moving-bleach dynamics, the majority of cells still showed recovery-in-place dynamics. This implies that there are additional controls to MamK dynamics in AMB-1 that are left to be discovered.

We further demonstrated that the interaction between McaA and McaB is correlated to changes in MamK dynamics. When the McaA-McaB interaction is removed through protein truncation or localization changes, MamK dynamics shift such that there are more cells exhibiting the moving-bleach-spot dynamics. This led us to hypothesize that MamK dynamics are locally controlled based on where McaAB interactions occur. McaB is at the crystal-containing magnetosomes in WT AMB-1 under normal biomineralization conditions, while McaA localizes from cell pole to pole along the positive curvature in a dashed pattern in all mutants and growth conditions tested (10). Previous co-localization studies of McaA-Halo and McaB-GFP in WT AMB-1 showed that McaB-GFP tends to localize within the gaps of the McaA-Halo signal (10). The inverted localization pattern suggests that McaA and McaB only interact at the interface of their territories along the magnetosome chain. Alternatively, the inverted localization pattern could be a result of dynamic McaA-McaB-MamK activity that results in minimized McaA-McaB interactions. In either case, there is a confinement of McaA-McaB interactions, which, in turn, generates localized zones of MamK regulation.

Altogether, we propose the following model for magnetosome chain organization in AMB-1 (Fig. 7B). McaB is recruited to a magnetosome that has begun biomineralization. This increases the proximity of McaB to the positive curvature membrane-localized McaA and MamK filaments at the magnetosome chain, allowing for protein-protein interactions. The interactions between the three proteins activate McaA and McaB to alter MamK filament directionality locally, which consequently moves the attached magnetosomes. The shifting of these crystal-containing magnetosomes causes some to join into a subchain and others to dislodge from an existing subchain. This, in turn, opens up space between crystal-containing magnetosomes for new, empty magnetosomes to form. The resulting magnetosome chain, therefore, has subchains of crystal-containing magnetosomes that are interrupted by empty magnetosomes.

Several pieces of this model remain to be resolved. First, are there other proteins involved in patterning magnetosomes in AMB-1? Though we have presented evidence that McaA and McaB directly interact with MamK, we could not prove a direct interaction between McaA and McaB *in vitro*. This leaves the possibility that there are other protein contributors to magnetosome organization not identified here. Second, we still do not know how MamK is altered by McaAB at the biochemical level. Possibilities include changes in filament polarity, sites of polymerization, or ATPase activity. Lastly, our model relies on McaAB interactions to specifically occur at the crystal-containing magnetosomes, which we have not demonstrated here. Future work should aim to address these unknowns.

### Changing MamK dynamics as a strategy to diversify magnetosome chain organization

Across MTB, we find diverse types of magnetosome chains. For example, there are species with multiple chains that are parallel or, strikingly, perpendicular to each other (34, 35). How did different types of magnetosome chain arrangements emerge across MTB species? One way is to change the machinery that controls magnetosome positioning. In *Desulfovibrio magneticus* RS-1, for example, a second bacterial actin, Mad28, and a suite of coiled-coil proteins are required for magnetosome chain assembly and organization in addition to MamK (11). Our work here reveals a possible second strategy where MamK is regulated to produce different magnetosome chains. This serves as a possible explanation for the difference in the magnetosome chain patterning between AMB-1 and its close relative *M. gryphiswaldense* MSR-1 (MSR-1). Both organisms use MamK, MamY, and MamJ to build a magnetosome chain, yet WT MSR-1 has a magnetosome chain with all crystal-containing magnetosomes at the chain center, just like AMB-1 Δ*mcaAB* (9, 17). Furthermore, in MSR-1, MamK polymerization has been reported to occur at the cell poles, thus pushing the MamK filaments towards midcell, resulting in a moving-bleach-spot dynamic when observed with FRAP (17). The ability to produce a singular crystal chain is not a unique property of MSR-1 MamK, since MSR-1 Δ*mamK* expressing AMB-1 *mamK* produces a similar magnetosome chain observed by TEM (36). Collectively, it seems likely that the difference in magnetosome chain patterning between AMB-1 and MSR-1 is due to different MamK behavior mediated by McaA and McaB. This case might be one example of a broader mechanism underlying organization diversification of magnetosomes and other subcellular structures in bacteria.

### McaA and McaB—bacterial actin-binding proteins?

Eukaryotic cells are known to have many actin-binding proteins that regulate all aspects of actin’s properties, ranging from capping proteins that prevent further actin polymerization to cross-linking proteins that stabilize higher-order filament structures (37). The massive collection of actin-binding proteins allows actin to be a multi-functional filament that can have different architectures and dynamics depending on the subset of actin-binding proteins that it is interacting with. Compared to the impressive repertoire of eukaryotic actin-binding proteins characterized, the discovery of analogous proteins in bacterial systems has lagged behind (38). One aspect is the lack of conservation between eukaryotic and bacterial actin-binding proteins, which is largely driven by the divergence of actin filament structure across bacterial actins (38, 39), hindering the computational identification of potential regulators. McaA and McaB interaction with MamK, and MamK’s behavioral change in their absence, opens the possibility that McaA and McaB are bacterial actin-binding proteins. Further studies on the biochemical mechanism of McaAB influence on MamK behavior would not only reveal whether they are true actin-binding proteins, but also serve as an example that will guide discoveries to other bacterial actin regulators.

## MATERIALS AND METHODS

### Bacterial strains and growth

*Magnetospirillum magneticum* sp. AMB-1 was cultured as previously described (10). Briefly, AMB-1 was grown on MG medium supplemented with 30 μM ferric malate and 1× Wolfe’s vitamin solution. A solid medium is prepared by adding 0.7% agar before autoclaving. AMB-1 colonies grown on MG plates were used to inoculate 1.5 mL liquid MG in 1.5 mL microcentrifuge tubes and then grown at 30°C for 2–3 days. These stock cultures were maintained at room temperature for a maximum duration of 2 weeks and diluted 100-fold into fresh MG medium for growth and *C*_mag_ measurements, TEM, and fluorescence microscopy. AMB-1 growth for co-IP experiments is detailed in a different section. Unless indicated otherwise, AMB-1 was cultured in glass culture tubes at 30°C in 10% oxygen without shaking for 1–2 days in a microaerobic glove box. If anaerobic medium was used, the medium was prepared by bubbling MG with N_2_ gas for at least 10 min before autoclaving in sealed Balch tubes. Ferric malate, Wolfe’s vitamin solution, and inoculum were introduced through a sterile needle and syringe. When needed, kanamycin was added for a final concentration of 7 μg/mL and 10 μg/mL for liquid and solid medium, respectively.

OD and coefficient of magnetism (*C*_mag_) readings for AMB-1 were taken on a Spectronic 20D+ at 400 nm. *C*_mag_ was determined by taking OD_400_ readings with a stir bar magnet held parallel and perpendicular to the sample. *C*_mag_ was calculated by taking the ratio of the two OD_400_ readings. A *C*_mag_ of 1 indicates no magnetic alignment.

*Escherichia coli* DH5ɑ, WM3064, and BL21 were grown in standard LB medium at 37°C, with 50 μg/mL kanamycin, 25 μg/mL chloramphenicol, 100 μg/mL carbenicillin, and/or 5 μM diaminopimelic acid (DAP) when needed, unless stated otherwise. The growth of *E. coli* strains used for bacterial two-hybrids is explained in a different section.

### Non-biomineralization conditions

All non-biomineralization cultures were grown in MG medium without the ferric malate solution in glassware treated overnight with 30 mM oxalic acid and then rinsed with MilliQ water three times to remove residual iron. To reduce existing magnetite in the AMB-1 cell, AMB-1 cultures were passaged in 10 mL non-biomineralization MG in acid-washed tubes three times, where each passage received a 100 μL inoculum of the previous passage and incubated for 2 days microaerobically at 30°C. The final culture was inoculated at a 1:100 dilution and grown at 30°C with constant stirring in the microaerobic glove box to incorporate 10% oxygen to further inhibit biomineralization.

### Genetic manipulation

All strains and plasmids used in this study can be found in the supplemental material. Plasmids used to express genes in AMB-1 or *E. coli* were cloned into *E. coli* DH5ɑ as previously described (10), with a few adjustments. Oligonucleotides were purchased from Integrated DNA Technologies, and PCR was done using Q5 High-Fidelity DNA Polymerase (New England Biolabs). Constructed plasmids were sequence-verified through the UC Berkeley DNA sequencing facility or Plasmidsaurus (www.plasmidsaurus.com). Briefly, to generate deletions in AMB-1, a suicide plasmid carrying two homologous regions flanking the target gene was transformed into *E. coli* WM3064. The plasmid was introduced into AMB-1 through conjugation, and then kanamycin and 2% sucrose were used to select for the first and second recombination events, respectively. Deletions were PCR amplified and sequence verified.

### Cell lysis and *in vivo* co-immunoprecipitation

Glass bottles of 2 L with 2 L of MG were inoculated with two 10 mL seed cultures of AMB-1 grown microaerobically for 2 days. These 2 L cultures were incubated for 2 days at 30°C in 10% oxygen without shaking. If applicable, 10 mL of the culture was taken to determine *C*_mag_ and perform microscopy. To harvest, cells were centrifuged in 500 mL centrifuge bottles at 11,000 *× g* with a JA-10 rotor and Beckman J2-21M centrifuge or GS-3 rotor and Sorvall RC-5B centrifuge. Cells were resuspended in 3–5 mL medium and transferred to a 15 mL Falcon tube. The residual medium was removed through a final centrifugation at 8000 × *g* for 6 min. Cell pellet mass was recorded, flash frozen in liquid nitrogen, and stored at −80°C until use. AMB-1 was subject to chemical lysis as previously reported (40). Briefly, cell pellets were resuspended in 1 mL lysis buffer A (10 mM Tris-HCl, 150 mM NaCl, 0.5 mM EDTA, 0.5% NP-40, pH 7.5, supplemented with 2 μg/mL pepstatin A, 2 μg/mL leupeptin, 2 mM PMSF, and 0.5 mg/mL lysozyme) and incubated at RT for 15 min without rotation. 3 mL of lysis buffer B (20 mM HEPES-KOH, 50 mM NaCl, 1.25 mM CaCl_2_, pH 7.5 with 2 mM DTT, and 5 μg/mL DNase I) was added, and samples were incubated at 4°C while rotating end-to-end for 45 min. Samples were then centrifuged at 14,600 × *g* for 30 min at 4°C. The supernatant was collected into a new 15 mL Falcon tube and either used for co-IP immediately or flash frozen in liquid nitrogen and stored at −80°C until use.

For co-IPs to be evaluated by Western blot, the volume of lysate used was normalized according to the cell pellet mass harvested and then diluted twofold with wash buffer (10 mM Tris-HCl, 150 mM NaCl, pH 7.4). 20 μL of GFP-Trap agarose beads (ChromoTek) were equilibrated three times by resuspending in 500 μL Wash Buffer, followed by centrifugation. All centrifugation steps here were carried out at 2,500 × *g* at 4°C, unless indicated otherwise. The diluted lysate was applied to the equilibrated beads in a 15 mL Falcon tube and rotated end-to-end for 1 h at 4°C. The solution was centrifuged for 6 min. The beads were transferred to a 1.5 mL microcentrifuge tube and washed three times with 500 μL wash buffer, centrifuged for 3 min between each wash. To elute, the beads were resuspended in 100 μL of 2× Laemmli sample buffer and incubated for 10 min at 95°C. The samples were centrifuged for 3 min, and the supernatant was collected as the elution.

The co-IP protocol was adjusted for samples to be sent for mass spectrometry. Ten 2L cultures were pooled (20 L total). Cell lysis was done with 5 mL lysis buffer A and 15 mL lysis buffer B in a 50 mL Falcon tube. The lysate was clarified by being split evenly into six 15 mL Falcon tubes before centrifugation to minimize debris contamination. 30 μL of the GFP-Trap agarose bead slurry was used for the co-IP. Elution was done by resuspending the samples in 50 μL of 200 mM glycine, pH 2.5, and vigorously pipetting up and down for 30 s. The sample was centrifuged at 2,500 × *g* for 3 min at 4°C. The supernatant was transferred to a new tube and neutralized with 5 μL of 1M Tris, pH 10.4. The beads were then eluted a second time to increase protein abundance.

### Mass spectrometry

Proteins were precipitated using trichloroacetic acid (TCA) precipitation. 100% TCA was added to the co-IP elution for a final TCA concentration of 20%. Samples were incubated on ice for 1 h and then centrifuged at 16,000 × *g* for 10 min at 4°C. The protein pellet was washed three times with cold 100 μL 0.01M HCL in 90% acetone and then allowed to dry at RT. Protein pellet mass was measured before proceeding. Precipitated protein samples were resuspended in 80 μL 100 mM Tris-HCl, 8 M urea, pH 8.5. TCEP (100 mM) was added for a final concentration of 5 mM and then incubated at RT for 20 min. Fresh 500 mM iodoacetamide was added for a 10 mM final concentration and then incubated at RT for 15 min in the dark. Samples were diluted fourfold using 100 mM Tris-HCl, pH 8.5. Filter-sterilized 100 mM CaCl_2_ was added for a final concentration of 1 mM before adding 1 μL of 0.5 μg/μL sequence-grade modified trypsin (Promega). Samples were incubated overnight at 37°C in the dark, and then formic acid was added to a 5% final concentration. Samples were then desalted using C18 Spec tips (Agilent). The Spec tip was washed with 200 μL HPLC-grade MeOH, followed by three washes with 20 μL 5% acetonitrile/5% formic acid. The sample was pushed through the tip, washed three times with 200 μL 5% acetonitrile/5% formic acid, eluted with two passes of 100 μL 80% acetonitrile/5% formic acid, and then dried.

Mass spectrometry was performed at the Proteomics/Mass Spectrometry Laboratory at the University of California, Berkeley. A nano LC column was packed in a 100-μm inner diameter glass capillary with an integrated pulled emitter tip. The column consisted of 10 cm of Polaris C18 5 μm packing material (Varian). The column was loaded and conditioned using a pressure bomb. The column was then coupled to an electrospray ionization source mounted on a Thermo-Fisher LTQ XL linear ion trap mass spectrometer. An Agilent 1200 HPLC equipped with a split line so as to deliver a flow rate of 1 μL/min was used for chromatography. Peptides were eluted with a 90-min gradient from 100% buffer A to 60% buffer B. Buffer A was 5% acetonitrile/0.02% heptafluorobutyric acid (HBFA); buffer B was 80% acetonitrile/0.02% HBFA. Collision-induced dissociation and electron transfer dissociation spectra were collected for each *m/z*. Protein identification, quantification, and analysis were done with Integrated Proteomics Pipeline-IP2 (Bruker Scientific LLC, Billerica, MA, http://www.bruker.com) using ProLuCID/Sequest (41), DTASelect2 (42, 43), and Census (44, 45). Spectrum raw files were extracted into ms1 and ms2 files from raw files using RawExtract 1.9.9 (http://fields.scripps.edu/downloads.php) 10, and the tandem mass spectra were searched against the MIT database (43, 46). Proteins encoded in AMB-1 magnetosome islet (MIS) were downloaded from NCBI on 1 September 2022 and included for analysis.

### Western blotting

Western blots were done with protein samples separated via SDS-PAGE using Mini-PROTEAN TGX Stain-Free (BioRad). Prior to transfer, gels were imaged for total protein content using Stain-Free imaging. The primary antibodies and their dilutions used in this study are as follows: anti-GFP (Abcam ab 6556, 1:2,500 and Invitrogen GF28R, 1:2,500), anti-Halotag (Promega G921A, 1:1,000), and anti-Flag (Sigma F3165, 1:2,500). The secondary antibodies and their dilutions are as follows: anti-mouse goat HRP conjugate (Invitrogen A24512, 1:5,000), anti-rabbit goat HRP conjugate (Biorad, 170-5046, 1:10,000). Blots were developed using Western Lighting Plus-ECL (PerkinElmer) and imaged with the BioRad ChemiDoc MP Imaging System. All full-sized blots can be found in the Source Data file.

### Epifluorescence microscopy

Ten milliliters of AMB-1 was microaerobically grown for 2 days at 30°C until mid-to-late exponential phase (OD_400_ of 0.200–0.280). This culture (1–1.5 mL) was centrifuged for 4 min at 8000 × *g*. The pellet was resuspended in 10 μL of residual medium, and 0.7 μL was applied to a glass slide. Imaging was done on a Zeiss Axio Observer Inverted microscope with Zen software (Zeiss), and images were handled using Fiji. To score images for localization, images were randomized using the Cell Counter plugin.

### Fluorescence recovery after photobleaching

AMB-1 cultures (10 mL) were grown microaerobically until OD_400_ reached ~0.1. The cultures were concentrated to 10 μL, of which 0.7 μL was applied between the bottom of a glass-bottom dish (MatTek) and a450 μL 2% agarose gumdrop that minimizes cell movement. Cells were imaged on an inverted Carl Zeiss LSM880 FCS laser scanning confocal microscope with an objective lens Plan-Apochromat 100×/1.40 oil DIC at RT. Cells were imaged with a 488 nm laser at 3% power every 30 s for 15 min with Definite Focus autofocusing (Zeiss). At *t* = 1 min, a small region of the cell was photobleached with the 488 nm laser at 100% power. Images were acquired through LSM880 Zen software (Zeiss). Fiji was used to analyze the images and generate kymographs.

### Transmission electron microscopy

AMB-1 cells to be used for TEM were grown in 10 mL cultures microaerobically for 2 days at 30°C. The culture (1–1.5 mL) was concentrated to 10 μL and applied to a Formvar/Carbon 300 Mesh copper grid (Electron Microscopy Sciences) that has been glow-discharged with Pelco Easiglow. After a 5-min incubation at RT, excess cells were removed by washing the grid three times in MilliQ water. Cells were imaged on an FEI Tecnai 12 transmission electron microscope with an accelerating voltage of 120 kV. Images were captured using a Rio 16 4K CMOS camera and Gatan Digital Micrograph software. Grids carrying cells to be used for scoring were randomized before imaging and scoring.

### Protein purification

To purify McaA and McaB, the predicted cytoplasmic side of both proteins was tagged and then expressed in BL21 *E. coli*. McaA (aa400–776) was N-terminally tagged with a Strep-tag, and McaB (aa 28–219) was N-terminally tagged with MBP. MBP was also expressed from BL21. BL21 harboring the recombinant protein expression plasmids was inoculated as a 1:100 dilution from an overnight seed culture grown in 2× YT without inducers and grown at 37°C shaking at 200 rpm. When OD_600_ reached ~0.5, expression of Strep-McaA and MBP was induced with 0.1 mM IPTG, and MBP-McaB expression was induced with 0.5 mM IPTG. Cultures were incubated for another 3–4 h at 37°C, shaking at 200 rpm. Cells were harvested at 10,800 × *g* for 20 min at 4°C, flash frozen with liquid nitrogen, and stored at −80°C until use. To lyse, cell pellets were resuspended in 35 mL 10 mM Tris-HCl, 150 mM NaCl, pH 7.4, supplemented with 1 μg/mL pepstatin A, 2 μg/mL leupeptin, and 1 mM PMSF, and lysed with a French press at 18,000 PSI. Lysate was clarified 14,600 × *g* for 30 min at 4°C, and stored at 4°C overnight. Lysates were filtered through a 0.22 μm PES syringe filter and applied to either a StrepTrap HP (GE Healthcare) or MBPTrap HP (Cytiva) column using an ÄKTA pure FPLC system with a 5 mL/min flow rate. Samples were washed with 20 column volumes (CV) with 10 mM Tris-HCl, 150 mM NaCl, pH 7.4, and eluted with 5 CV with either 10 mM Tris-HCl, 150 mM NaCl, 2.5 mM desthiobiotin, pH 7.4 for StrepTrap or 10 mM Tris-HCl, 150 mM NaCl, 10 mM maltose, pH 7.4 for MBPTrap. Fractions were evaluated with SDS-PAGE, and those containing the protein of interest were pooled and concentrated with Amicon Ultra 10K centrifugal filter units. Samples were further purified via size exclusion chromatography using a HiLoad 16/600 Superdex 75 pg column (GE Healthcare) with 10 mM Tris-HCl, 150 mM NaCl, pH 7.4. Fractions with pure proteins were flash frozen and stored at −80°C until use. Protein concentrations were determined using the Micro BCA Protein Assay Kit.

‏Untagged recombinant MamK was purified from *E. coli* using ammonium sulfate precipitation as previously described (20), with a few adjustments. After the second 20% ammonium sulfate cut, MamK samples are dialyzed into depolymerization buffer (10 mM CAPs, 10 mM EDTA, 2 mM DTT, pH 9.6) at 4°C for approximately 8 h. The sample was further purified using anion exchange chromatography (DEAE TOYOPEARL, 2.3 cm × 12 cm, Tosoh, Japan) and gel filtration chromatography (Superdex 200 Increase 10/300 GL, Cytiva, USA) with depolymerization buffer. The monomeric MamK fractions were pooled, and aliquots were stored at −80°C. For experimental use, purified MamK is exchanged into polymerization buffer (10 mM Tris-HCl, 25 mM KCl, 2 mM MgCl_2_, 1 mM DTT, pH 7.5) using a desalting spin column (Zaba Spin Desalting Column, Thermo Fisher, USA).

### Pelleting assay

Protein samples were diluted to 1 µM in polymerization buffer (10 mM Tris-HCl, pH 7.5, 25 mM KCl, 2 mM MgCl_2_, 1 mM DTT). Proteins in mixtures each had a concentration of 1 µM. Final concentration of 1 mM ATP was added if needed. Samples were incubated at 25°C for 10 min and then centrifuged at 100,000 × *g* for 30 min at 4°C. The supernatant and pellet fractions were recovered and analyzed by SDS-PAGE.

### *In vitro* pull down

Ten micrograms of each protein was mixed into a total of 500 μL of equilibration buffer (10 mM Tris-HCl, 150 mM NaCl, pH 7.4) and incubated at 4°C for 1 h with end-to-end mixing. All the following steps were carried out at 4°C. For negative controls, 10 μg of each protein was incubated separately. 20 μL Strep-tactin resin (IBA Lifesciences) was equilibrated by washing three times with 100 μL equilibration buffer and centrifuging at 2,500 × *g* for 3 min at 4°C between each wash. Protein samples were centrifuged at 16,000 × *g* for 5 min to remove any aggregates. The supernatant was added to the equilibrated Strep-tactin resin in a 1.5 mL microcentrifuge tube and incubated for 1 h while end-to-end mixing. The sample was centrifuged at 2,500 × *g* for 3 min, and the flow-through supernatant was removed. The resin was washed three times using 500 μL equilibration buffer and then eluted with 50 μL of 10 mM Tris-HCl, 150 mM NaCl, 2.5 mM desthiobiotin, pH 7.4, by pipetting vigorously for 30 s. The elution was collected by centrifuging for 3 min at 2,500 × *g*. Samples were evaluated with SDS-PAGE and Coomassie staining.

### Bacterial adenylate cyclase two-hybrid assay

*E. coli* DHM1 was transformed with two plasmids, each carrying either the T18 or T25 fusion, and incubated at 30°C overnight with 50 μg/mL kanamycin and 100 μg/mL carbenicillin for selection. A single colony was used to inoculate 80 μL LB with kanamycin, carbenicillin, and 0.5 mM IPTG in a 96-well plate, and incubated at 30°C overnight, shaking at 200 rpm. 3 μL of the culture was spotted onto M63 agar supplemented with 25 μg/mL kanamycin, 50 μg/mL carbenicillin, 0.5 mM IPTG, and 40 μg/mL X-gal and incubated at 30°C for 5–8 days before imaging with an iPhone 15 camera.

### Multiple sequence alignment

McaA homologs were first identified through a BlastP search of AMB-1 McaA on 27 December 2024. Homolog sequences with an expectation value below 1e-5 from this initial search were downloaded from NCBI on the same day. The homolog sequences were then aligned on 14 January 2025, using MAFFT (Version 7.511) using the iterative refinement algorithm L-INS-i (47). The alignment was visualized using Jalview (version 2.11.4.1) (48).

### McaA and McaB protein sequence analysis

McaA protein structure was predicted using AlphaFold3 on the Alphafold Server (https://alphafoldserver.com/) on 12 November 2022 (29). Deepcoil2 (https://toolkit.tuebingen.mpg.de/tools/deepcoil2) was used to predict coiled-coil domains, “a” position amino acids, and “d” position amino acids of McaB (28). The predicted domains and amino acids were mapped onto predicted McaB structures generated by AlphaFold3 on 20 October 2025. Protein structures were visualized using UCSF ChimeraX (49).

## Data Availability

The data from the IP-MS and FRAP experiments can be found in Data S2 and S3.

## SUPPLEMENTAL MATERIAL

Data S1 mbio.00276-26-s0001.pdf Figures S1-S14.

Data S2 mbio.00276-26-s0002.xlsx IP MS data.

Data S3 mbio.00276-26-s0003.xlsx Source data for FRAP experiments

Data S4 mbio.00276-26-s0004.xlsx Source data for Fig. 1-7.

Data S5 mbio.00276-26-s0005.xlsx Source data for Fig. S1-S7.

Data S6 mbio.00276-26-s0006.xlsx Source data for Fig. S8-S14.

Data S7 mbio.00276-26-s0007.xlsx Genetic tools.

Data S8 mbio.00276-26-s0008.xlsx Statistics.

Legends mbio.00276-26-s0009.pdf Legends for Data S2-S8.

ASM does not own the copyrights to Supplemental Material that may be linked to, or accessed through, an article. The authors have granted ASM a non-exclusive, world-wide license to publish the Supplemental Material files. Please contact the corresponding author directly for reuse.

## References

[1] Greening C, Lithgow T. 2020. Formation and function of bacterial organelles. Nat Rev Microbiol 18:677–689. doi:10.1038/s41579-020-0413-0 32710089

[2] Iancu CV, Morris DM, Dou Z, Heinhorst S, Cannon GC, Jensen GJ. 2010. Organization, structure, and assembly of α-carboxysomes determined by electron cryotomography of intact cells. J Mol Biol 396:105–117. doi:10.1016/j.jmb.2009.11.019 19925807 PMC2853366

[3] Beeby M, Cho M, Stubbe J, Jensen GJ. 2012. Growth and localization of polyhydroxybutyrate granules in Ralstonia eutropha. J Bacteriol 194:1092–1099. doi:10.1128/JB.06125-11 22178974 PMC3294789

[4] Dobro MJ, Oikonomou CM, Piper A, Cohen J, Guo K, Jensen T, Tadayon J, Donermeyer J, Park Y, Solis BA, Kjær A, Jewett AI, McDowall AW, Chen S, Chang Y-W, Shi J, Subramanian P, Iancu CV, Li Z, Briegel A, Tocheva EI, Pilhofer M, Jensen GJ. 2017. Uncharacterized bacterial structures revealed by electron cryotomography. J Bacteriol 199:e00100-17. doi:10.1128/JB.00100-17 28607161 PMC5553035

[5] Bazylinski DA, Frankel RB. 2004. Magnetosome formation in prokaryotes. Nat Rev Microbiol 2:217–230. doi:10.1038/nrmicro842 15083157

[6] Blakemore R. 1975. Magnetotactic bacteria. Science 190:377–379. doi:10.1126/science.170679 170679

[7] Komeili A, Vali H, Beveridge TJ, Newman DK. 2004. Magnetosome vesicles are present before magnetite formation, and MamA is required for their activation. Proc Natl Acad Sci USA 101:3839–3844. doi:10.1073/pnas.0400391101 15004275 PMC374331

[8] Paulus A, Ahrens F, Schraut A, Hofmann H, Schiller T, Sura T, Becher D, Uebe R. 2024. MamF-like proteins are distant Tic20 homologs involved in organelle assembly in bacteria. Nat Commun 15:10657. doi:10.1038/s41467-024-55121-0 39653729 PMC11628618

[9] Scheffel A, Gruska M, Faivre D, Linaroudis A, Plitzko JM, Schüler D. 2006. An acidic protein aligns magnetosomes along a filamentous structure in magnetotactic bacteria. Nature 440:110–114. doi:10.1038/nature04382 16299495

[10] Wan J, Monteil CL, Taoka A, Ernie G, Park K, Amor M, Taylor-Cornejo E, Lefevre CT, Komeili A. 2022. McaA and McaB control the dynamic positioning of a bacterial magnetic organelle. Nat Commun 13:5652. doi:10.1038/s41467-022-32914-9 36163114 PMC9512821

[11] Russell VV, Iavarone AT, Ozyamak E, Grant C, Komeili A. 2025. A network of coiled-coil and actin-like proteins controls the cellular organization of magnetosome organelles in deep-branching magnetotactic bacteria. Nat Commun 16:11453. doi:10.1038/s41467-025-66326-2 41381523 PMC12748827

[12] Pfeiffer D, Schüler D. 2020. Quantifying the benefit of a dedicated “magnetoskeleton” in bacterial magnetotaxis by live-cell motility tracking and soft agar swimming assay. Appl Environ Microbiol 86:e01976-19. doi:10.1128/AEM.01976-19 31732570 PMC6974628

[13] Komeili A, Li Z, Newman DK, Jensen GJ. 2006. Magnetosomes are cell membrane invaginations organized by the actin-like protein MamK. Science 311:242–245. doi:10.1126/science.1123231 16373532

[14] Toro-Nahuelpan M, Giacomelli G, Raschdorf O, Borg S, Plitzko JM, Bramkamp M, Schüler D, Müller F-D. 2019. MamY is a membrane-bound protein that aligns magnetosomes and the motility axis of helical magnetotactic bacteria. Nat Microbiol 4:1978–1989. doi:10.1038/s41564-019-0512-8 31358981 PMC6817358

[15] Katzmann E, Scheffel A, Gruska M, Plitzko JM, Schüler D. 2010. Loss of the actin-like protein MamK has pleiotropic effects on magnetosome formation and chain assembly in Magnetospirillum gryphiswaldense. Mol Microbiol 77:208–224. doi:10.1111/j.1365-2958.2010.07202.x 20487281

[16] Draper O, Byrne ME, Li Z, Keyhani S, Barrozo JC, Jensen G, Komeili A. 2011. MamK, a bacterial actin, forms dynamic filaments in vivo that are regulated by the acidic proteins MamJ and LimJ. Mol Microbiol 82:342–354. doi:10.1111/j.1365-2958.2011.07815.x 21883528 PMC3540106

[17] Toro-Nahuelpan M, Müller FD, Klumpp S, Plitzko JM, Bramkamp M, Schüler D. 2016. Segregation of prokaryotic magnetosomes organelles is driven by treadmilling of a dynamic actin-like MamK filament. BMC Biol 14:88. doi:10.1186/s12915-016-0290-1 27733152 PMC5059902

[18] Cornejo E, Subramanian P, Li Z, Jensen GJ, Komeili A. 2016. Dynamic remodeling of the magnetosome membrane is triggered by the initiation of biomineralization. mBio 7:e01898-15. doi:10.1128/mBio.01898-15 PMC4791847 26884433

[19] Taoka A, Kiyokawa A, Uesugi C, Kikuchi Y, Oestreicher Z, Morii K, Eguchi Y, Fukumori Y. 2017. Tethered magnets are the key to magnetotaxis: direct observations of Magnetospirillum magneticum AMB-1 show that MamK distributes magnetosome organelles equally to daughter cells. mBio 8:e00679-17. doi:10.1128/mBio.00679-17 28790202 PMC5550748

[20] Ozyamak E, Kollman J, Agard DA, Komeili A. 2013. The bacterial actin MamK: in vitro assembly behavior and filament architecture. J Biol Chem 288:4265–4277. doi:10.1074/jbc.M112.417030 23204522 PMC3567678

[21] Löwe J, He S, Scheres SHW, Savva CG. 2016. X-ray and cryo-EM structures of monomeric and filamentous actin-like protein MamK reveal changes associated with polymerization. Proc Natl Acad Sci USA 113:13396–13401. doi:10.1073/pnas.1612034113 27821762 PMC5127371

[22] Pan Y, Kikuchi Y, Saito T, Fukumori Y, Taoka A. 2025. Visualizing the dynamic polymerization of the bacterial actin-like cytoskeleton for magnetic organelle positioning. Sci Rep 15:44508. doi:10.1038/s41598-025-28026-1 41444272 PMC12738573

[23] Scheffel A, Schüler D. 2007. The acidic repetitive domain of the Magnetospirillum gryphiswaldense MamJ protein displays hypervariability but is not required for magnetosome chain assembly. J Bacteriol 189:6437–6446. doi:10.1128/JB.00421-07 17601786 PMC1951895

[24] Garner EC, Campbell CS, Weibel DB, Mullins RD. 2007. Reconstitution of DNA segregation driven by assembly of a prokaryotic actin homolog. Science 315:1270–1274. doi:10.1126/science.1138527 17332412 PMC2851738

[25] Polka JK, Kollman JM, Mullins RD. 2014. Accessory factors promote AlfA-dependent plasmid segregation by regulating filament nucleation, disassembly, and bundling. Proc Natl Acad Sci USA 111:2176–2181. doi:10.1073/pnas.1304127111 24481252 PMC3926056

[26] Petek-Seoane NA, Rodriguez J, Derman AI, Royal SG, Lord SJ, Lawrence R, Pogliano J, Mullins RD. 2024. Polymer dynamics of Alp7A reveals how two critical concentrations govern assembly of dynamically unstable actin-like proteins. Mol Biol Cell 35:ar145. doi:10.1091/mbc.E23-11-0440 39320937 PMC11617094

[27] Burgess JG, Kawaguchi R, Sakaguchi T, Thornhill RH, Matsunaga T. 1993. Evolutionary relationships among Magnetospirillum strains inferred from phylogenetic analysis of 16S rDNA sequences. J Bacteriol 175:6689–6694. doi:10.1128/jb.175.20.6689-6694.1993 7691800 PMC206781

[28] Ludwiczak J, Winski A, Szczepaniak K, Alva V, Dunin-Horkawicz S. 2019. DeepCoil-a fast and accurate prediction of coiled-coil domains in protein sequences. Bioinformatics 35:2790–2795. doi:10.1093/bioinformatics/bty1062 30601942

[29] Abramson J, Adler J, Dunger J, Evans R, Green T, Pritzel A, Ronneberger O, Willmore L, Ballard AJ, Bambrick J, et al.. 2024. Accurate structure prediction of biomolecular interactions with AlphaFold 3. Nature 630:493–500. doi:10.1038/s41586-024-07487-w 38718835 PMC11168924

[30] Murat D, Quinlan A, Vali H, Komeili A. 2010. Comprehensive genetic dissection of the magnetosome gene island reveals the step-wise assembly of a prokaryotic organelle. Proc Natl Acad Sci USA 107:5593–5598. doi:10.1073/pnas.0914439107 20212111 PMC2851823

[31] Rioux J-B, Philippe N, Pereira S, Pignol D, Wu L-F, Ginet N. 2010. A second actin-like MamK protein in Magnetospirillum magneticum AMB-1 encoded outside the genomic magnetosome island. PLoS One 5:e9151. doi:10.1371/journal.pone.0009151 20161777 PMC2818848

[32] Arakaki A, Kikuchi D, Tanaka M, Yamagishi A, Yoda T, Matsunaga T. 2016. Comparative subcellular localization analysis of magnetosome proteins reveals a unique localization behavior of Mms6 protein onto magnetite crystals. J Bacteriol 198:2794–2802. doi:10.1128/JB.00280-16 27481925 PMC5038011

[33] Bickley CD, Wan J, Komeili A. 2024. Intrinsic and extrinsic determinants of conditional localization of Mms6 to magnetosome organelles in Magnetospirillum magneticum AMB-1. J Bacteriol 206:e00008-24. doi:10.1128/jb.00008-24 38819153 PMC11332177

[34] Schüler D. 2008. Genetics and cell biology of magnetosome formation in magnetotactic bacteria. FEMS Microbiol Rev 32:654–672. doi:10.1111/j.1574-6976.2008.00116.x 18537832

[35] Bidaud CC, Monteil CL, Menguy N, Busigny V, Jézéquel D, Viollier É, Travert C, Skouri-Panet F, Benzerara K, Lefevre CT, Duprat É. 2021. Biogeochemical niche of magnetotactic cocci capable of sequestering large polyphosphate inclusions in the anoxic layer of the lake pavin water column. Front Microbiol 12:789134. doi:10.3389/fmicb.2021.789134 35082768 PMC8786505

[36] Awal RP, Müller FD, Pfeiffer D, Monteil CL, Perrière G, Lefèvre CT, Schüler D. 2023. Experimental analysis of diverse actin-like proteins from various magnetotactic bacteria by functional expression in Magnetospirillum gryphiswaldense. mBio 14:e01649-23. doi:10.1128/mbio.01649-23 37823629 PMC10653835

[37] Pollard TD. 2016. Actin and actin-binding proteins. Cold Spring Harb Perspect Biol 8:a018226. doi:10.1101/cshperspect.a018226 26988969 PMC4968159

[38] Charles-Orszag A, Petek-Seoane NA, Mullins RD. 2024. Archaeal actins and the origin of a multi-functional cytoskeleton. J Bacteriol 206:e0034823. doi:10.1128/jb.00348-23 38391233 PMC10955848

[39] Ozyamak E, Kollman JM, Komeili A. 2013. Bacterial actins and their diversity. Biochemistry 52:6928–6939. doi:10.1021/bi4010792 24015924 PMC4318550

[40] Hershey DM, Browne PJ, Iavarone AT, Teyra J, Lee EH, Sidhu SS, Komeili A. 2016. Magnetite biomineralization in Magnetospirillum magneticum is regulated by a switch-like behavior in the HtrA protease MamE. J Biol Chem 291:17941–17952. doi:10.1074/jbc.M116.731000 27302060 PMC5016182

[41] Xu T, Park SK, Venable JD, Wohlschlegel JA, Diedrich JK, Cociorva D, Lu B, Liao L, Hewel J, Han X, Wong CCL, Fonslow B, Delahunty C, Gao Y, Shah H, Yates JR. 2015. ProLuCID: an improved SEQUEST-like algorithm with enhanced sensitivity and specificity. J Proteomics 129:16–24. doi:10.1016/j.jprot.2015.07.001 26171723 PMC4630125

[42] Cociorva D, L. Tabb D, Yates JR. 2006. Validation of tandem mass spectrometry database search results using DTASelect. Curr Protocols Bioinform 16:13. doi:10.1002/0471250953.bi1304s16 18428785

[43] Tabb DL, McDonald WH, Yates JR. 2002. DTASelect and Contrast: tools for assembling and comparing protein identifications from shotgun proteomics. J Proteome Res 1:21–26. doi:10.1021/pr015504q 12643522 PMC2811961

[44] Park SK, Venable JD, Xu T, Yates JR. 2008. A quantitative analysis software tool for mass spectrometry-based proteomics. Nat Methods 5:319–322. doi:10.1038/nmeth.1195 18345006 PMC3509211

[45] Park SKR, Aslanian A, McClatchy DB, Han X, Shah H, Singh M, Rauniyar N, Moresco JJ, Pinto AFM, Diedrich JK, Delahunty C, Yates JR III. 2014. Census 2: isobaric labeling data analysis. Bioinformatics 30:2208–2209. doi:10.1093/bioinformatics/btu151 24681903 PMC4155478

[46] Eng JK, McCormack AL, Yates JR. 1994. An approach to correlate tandem mass spectral data of peptides with amino acid sequences in a protein database. J Am Soc Mass Spectrom 5:976–989. doi:10.1016/1044-0305(94)80016-2 24226387

[47] Katoh K, Standley DM. 2013. MAFFT multiple sequence alignment software version 7: improvements in performance and usability. Mol Biol Evol 30:772–780. doi:10.1093/molbev/mst010 23329690 PMC3603318

[48] Waterhouse AM, Procter JB, Martin DMA, Clamp M, Barton GJ. 2009. Jalview Version 2--a multiple sequence alignment editor and analysis workbench. Bioinformatics 25:1189–1191. doi:10.1093/bioinformatics/btp033 19151095 PMC2672624

[49] Meng EC, Goddard TD, Pettersen EF, Couch GS, Pearson ZJ, Morris JH, Ferrin TE. 2023. UCSF ChimeraX: Tools for structure building and analysis. Protein Sci 32:e4792. doi:10.1002/pro.4792 37774136 PMC10588335

