# Supplementary material for: Magnetosome organelles are organized through interactions between McaA and McaB that alter the dynamics of the bacterial actin-like protein MamK
Source: mBio. 2026 Apr 3;17(5):e00276-26. doi: 10.1128/mbio.00276-26 (PMC13170167; doi:10.1128/mbio.00276-26)
Supplement: Legends — for Data S2-S8. [file mbio.00276-26-s0009.pdf]

Supplemental Data Legends.

Supplemental Data 2. Source data for mass spectrometry data for immunoprecipitation experiments with McaA-GFP, McaB-GFP, and GFP constructs.

Supplemental Data 3. Source data for FRAP experiments.

Supplemental Data 4. Source data for main figures.

Supplemental Data 5. Source data for supplemental figures S1-S7.

Supplemental Data 6. Source data for supplemental figures S8-S14.

Supplemental Data 7. Lists of strains, plasmids, and primers used in this study.

Supplemental Data 8. List of statistical methods and values used in this study.
